# *PRCC-TFE3* dual-fusion FISH assay: A new method for identifying *PRCC-TFE3* renal cell carcinoma in paraffin-embedded tissue

**DOI:** 10.1371/journal.pone.0185337

**Published:** 2017-09-26

**Authors:** Lei Xiong, Xiancheng Chen, Ning Liu, Zhen Wang, Baolei Miao, Weidong Gan, Dongmei Li, Hongqian Guo

**Affiliations:** 1 Department of Urology, Nanjing Drum Tower Hospital, Medical School of Nanjing University, Institute of Urology, Nanjing University, Nanjing, China; 2 Department of Critical Care Medicine, Nanjing Drum Tower Hospital, Medical School of Nanjing University, Nanjing, China; 3 Immunology and Reproductive Biology Laboratory & State Key Laboratory of Analytical Chemistry for Life Science, Medical School of Nanjing University, Nanjing, China; 4 Jiangsu Key Laboratory of Molecular Medicine, Nanjing University, Nanjing, China; National Institute of Health, UNITED STATES

## Abstract

*PRCC-TFE3* renal cell carcinoma (RCC) is one of the most common types of Xp11.2 translocation renal cell carcinoma (tRCC), of which the diagnosis mainly relies on reverse transcription-polymerase chain reaction (RT-PCR) or chromosomal analysis in fresh frozen samples. Herein, we developed a new dual-fusion fluorescence in situ hybridization (FISH) probe to succinctly identify *PRCC-TFE3* RCC in paraffin-embedded tissue. We immunohistochemically analyzed TFE3 and cathepsin K expression in 23 cases of Xp11.2 tRCC which had been confirmed by break-apart *TFE3* FISH probe. Next, the dual-fusion FISH assay was performed on these selected cases. Twenty typical cases of clear renal cell carcinoma and 20 cases of papillary renal cell carcinoma were collected as control groups. Seven cases were finally confirmed as *PRCC-TFE3* RCC by FISH detection, emerging dual-fusion signals, of which 2 cases were identified as *PRCC-TFE3* RCC by RT-PCR previously. All remaining cases were negative for the *PRCC-TFE3* rearrangement by FISH. The TFE3 immunohistochemistry was positive in 22/23 cases and the cathepsin K was positive in 16/23 cases. All 7 *PRCC-TFE3* RCCs showed positive cathepsin K immunoreactivity. Our results reveal that *PRCC-TFE3* dual-fusion FISH probe is an efficient and concise technique for diagnosing *PRCC-TFE3* RCC in paraffin-embedded tissue.

## Introduction

Xp11.2 translocation renal cell carcinoma (tRCC), a rare subtype of renal cell carcinoma (RCC), result from gene fusions involving the *TFE3* transcription factor gene[1], and it is included into the MiT family tRCCs in the recently published World Health Organization (WHO) classification of tumors of the urinary system[2]. Among renal carcinomas, Xp11.2 tRCCs comprise 20% to 75% of paediatric renal neoplasms [3] and about 1.5% of adult RCC cases [4]. Most common gene fusions in Xp11.2 tRCC are *TFE3* gene on Xp11.2 with *PRCC* at 1q21 and *TFE3* with *ASPL* at 17q25, which arise from the translocations t(X; 1) (p11.2; q21)[5] and t(X; 17) (p11.2; q25.3)[6]. Other less recurrent reported *TFE3* fusion partners include *SFPQ* (alias *PSF*), *NonO* [7], *CLTC* [8] and *RBM10* [9], resulting from t(X; 1) (p11.2; p34), inv(X) (p11.2q12), t(X; 17) (p11.2; q23), and inv(X) (p11.2p11.23), respectively. Some fusion partners, such as *PARP14* [10], *KHTFESRP* [11], *LUC7L3* [11] and *DVL2* [12], have only been identified in single patients.

Regarding diagnosis, when fresh frozen samples or viable tumor cells are available, reverse transcription-polymerase chain reaction (RT-PCR) or cytogenetic karyotypic analysis is highly sensitive and specific technique to confirm known gene alteration [13]. However, in archival formalin-fixed paraffin-embedded (FFPE) tissues, morphological characteristics and immunohistochemistry (IHC) are the diagnostic bases of Xp11.2 tRCC. Morphologically, distinctive features of Xp11.2 tRCC are papillary architecture composed of clear or eosinophilic cells and psammoma bodies [1]. However, Xp11.2 tRCCs often present with unusual morphology, which can mimic other types of RCC, and can also be mimicked by some other atypical tumors [14]. In contrast to most RCCs, Xp11.2 tRCC underexpress epithelial markers, but TFE3 and cathepsin K are always high expressed [14]. TFE3 is a ubiquitously expressed transcription factor which contains a basic helix-loop-helix region followed by a leucine zipper (bHLHzip) [15], but its normal level is generally undetectable by IHC. Once *TFE3* gene fusion occurs, the partners as strong promoters lead to overexpression of the fusion proteins which can be clearly detected immunohistochemically [14]. IHC with the polyclonal TFE3 antibody was regarded as a very sensitive and specific diagnostic method for Xp11.2 RCC previously[8], but over time increasing evidences have showed false-positive, false-negative, and equivocal results in *TFE3* IHC[16, 17]. Studies have found that high sensitivity and specificity is always achieved with a manual overnight labeling, but the automated method with short incubation time creates more false positives. However, in routine, immunohistochemistry in automated immunostainer with a 30 min incubation period is more common [8,18,19]. Cathepsin K is another possible immunohistochemical marker for MiT family tRCC[20]. It plays an important role in osteoclast function, whose expression can be mediated by aberrantly expressed microphthalmia transcription factors [21]. Among Xp11.2 RCCs, approximately 60% label for cathepsin K, while almost all conventional RCCs stain negative [20, 22]. Recently, break-apart fluorescence in situ hybridization (FISH) assay in FFPE archival sections is viewed as the optimal test for diagnosing Xp11.2 RCC for most laboratories [23] (However, the break-apart FISH assay is less useful than TFE3 immunohistochemistry in detecting some Xp11.2 RCCs, like the RBM10-TFE3 RCC which are associated with a subtle chromosome inversion [9].), while it is unable to identify the fusion partner of *TFE3* gene.

*PRCC-TFE3* RCC, one of the most common types of Xp11.2 tRCCs, is the first documented case of Xp11.2 tRCCs[24]. The breakpoint of the translocation in *PRCC-TFE3* RCC was cloned in 1996, and it was found that this translocation resulted in a fusion of the *TFE3* gene on the Xp11.2 to a novel *PRCC* gene on 1q21.2 [25–27]. The morphologic features of this tumor are slightly different from *ASPL-TFE3* RCC; however *PRCC-TFE3* RCC cannot be distinguished from other Xp11.2 tRCCs on these criteria alone [28]. Immunohistochemically, the expression of cathepsin K in *PRCC-TFE3* RCC is more frequent than *ASPL-TFE3* RCC, indicating biologic differences among subtypes of Xp11.2 tRCCs [14]. Although previous studies showed that *PRCC-TFE3* RCC presented at lower stage and less metastatic frequency than *ASPL-TFE3* RCC, metastatic disease was also found to occur in some *PRCC-TFE3* RCCs [14, 29]. Therefore, accurate, concise and early diagnosis of *PRCC-TFE3* RCC would benefit clinical management and research.

Up to now, it has been difficult to diagnose *PRCC-TFE3* RCC only relying on pathologic morphology, IHC, and break-apart FISH probe in FFPE archival tissue in clinical practice. Clinicopathologic data of *PRCC-TFE3* RCC and other subtypes of Xp11.2 tRCC are limited. In a previous study, we have demonstrated the value of a novel *ASPL-TFE3* dual-fusion FISH probe in confirming *ASPL* and *TFE3* gene fusion [30]. We report herein a *PRCC-TFE3* dual-fusion FISH assay for diagnosing *PRCC-TFE3* RCC and collect the clinical, pathological, and IHC features of the cases.

## Patients and Methods

### Case selection

A total of 23 cases of Xp11.2 tRCC (from January 2007 to February 2015) were included in this study, which were confirmed by break-apart *TFE3* FISH probe, and 6 Xp11.2 tRCCs (2 with *ASPL-TFE3* gene fusion, 2 with *PRCC-TFE3* gene fusion, 1 with *PSF-TFE3* gene fusion, and 1 with *CLTC-TFE3* gene fusion) were genetically confirmed by RT-PCR previously[30]. FFPE tissues were obtained from resection specimens from the files of the department of Pathology, Nanjing Drum Tower Hospital, Medical School of Nanjing University, and prepared for IHC and FISH assay. The clinicopathologic features including clinical manifestations, treatment strategies, pathological findings, clinical outcomes and follow-up information of Xp11.2 tRCC were recorded and evaluated. Twenty typical cases of clear renal cell carcinoma and 20 cases of papillary renal cell carcinoma were also collected as control groups. This study was approved by the Institutional Review Board of Nanjing Drum Tower Hospital. All the patients have signed informed written consents to have their medical record data used in research.

### Immunohistochemistry

Immunoreaction for TFE3 (prediluted, ZSGB-BIO, Beijing, China) and cathepsin K (Abcam, Cambridge, MA, USA) were performed in tumor tissue sections of all RCCs. Briefly, after deparaffinization, four-micrometer-thick paraffin-embedded sections were rehydrated using graded ethanol concentrations and treated in hydrogen peroxidase and absolute alcohol for 10 minutes at room temperature to block endogenous peroxidase activity. The tissue sections were subsequently incubated with the primary antibody against the TFE3 or cathepsin K protein for 40 minutes at 25°C. After tris-buffered saline rinses, the tissue was incubated using the secondary antibody for 30 minutes followed by diaminobenzidine for 5 minutes. Positive and negative controls were stained concurrently and showed appropriate immunostaining.

The immunohistochemical assessment for TFE3 and cathepsin K were evaluated as previously described [31, 32]. We assessed the cathepsin K and *TFE3* expression by the proportion or intensity of positive cells. For cathepsin K, a score was assigned to represent the estimated percentage of positive cells as follows: 0 or negative, <5% tumor cell positivity; + or focal, 5% to 10% tumor cell positivity; 2+ or moderate, 11% to 50% tumor cell positivity; and 3+ or strong, >50% tumor cell positivity. Moderately (2+) to strongly (3+) positive staining was considered positive result. For *TFE3*, we defined nuclear immunoreactivity that was apparent at low-power magnification as positive result. And these cases were subdivided into moderately (2+) and strongly (3+) positive on the basis of intensity.

### *PRCC-TFE3* dual-fusion FISH assay

FISH on the paraffin-embedded materials were performed with a probe consisting of 2 contigs that covered the entire *TFE3* gene on the short arm of the X chromosome and the *PRCC* gene on the long arm of chromosome 1. The contig on the X chromosome consisted of 7 BAC clones (CTD-2311N12, RP11-416B14, CTD-2522M13, CTD-2516D6, CTD-2312C1, CTD-2248C21 and RP11-959H17) labeled with fluorescein-12-dUTP as green fluorescein, and the contig on chromosome 1 consisted of 4 BAC clones (CTD-2534H6, RP11-1047J23, RP11-730I22 and CTD-2547N15) labeled with tetramethylrhodamine-5-dUTP as red fluorescein.

After deparaffinization and washing, three-micrometer-thick paraffin-embedded sections were rehydrated in 100%, 85%, and 70% ethanol in turn for 3 minutes and digested with 10μL pepsin (4 mg/mL, 0.02M HCl; Sigma-Aldrich, Beijing, China) at 37°C for 3 to 5 minutes, followed by subsequent dehydration. Then, the probe mixture was applied, and the slides were denatured at 85°C for 5 minutes and target DNA simultaneously, followed by hybridization overnight at 37°C. The slides were immersed in 2×SSC for 10 minutes and in 0.1% NP-40/2×SSC for 5 minutes at 37°C. The nuclei were counterstained with 4, 6-diamidino-2-phenylindole (DAPI). After hybridization, all slides were maintained at 4°C in the dark.

The slides were examined using an Olympus BX51TRF fluorescence microscope (Olympus, Tokyo, Japan) with the following three filters (DAPI/FITC/TexasRed) and the FISH analysis software (Imstar, Paris, France). For each case, a minimum of 100 nonoverlapping tumor cell nuclei were examined under fluorescence microscopy. Cells without the rearrangement presented split green and red signals (negative result), indicating intact Xp11 and 1q21. Dual fusion signal pattern (two fusion signals) was interpreted as the existence of reciprocal translocation of *PRCC* gene and *TFE3* gene (positive result). We considered a yellow or closely approximated green-red signal as a fusion signal. Clear FISH signals should be observed in >100 nonoverlapping nuclei for each case.

Normal renal tissues from 20 cases of non-PRCC-TFE3 renal cell carcinoma were randomly selected to determine the cutoff. We observed the signals of 50 nuclei in each case, and calculated the percentage of the cells with fusion signal. Subsequently, mean and standard deviation of the percentages were calculated. The cutoff is the sum of mean and three times of standard deviation. Using this method, a positive result was reported when >2% of the tumor nuclei showed the fusion-signal pattern. (http://dx.doi.org/10.17504/protocols.io.jdfci3n)

## Results

### Patients

The total 23 Xp11.2 tRCCs were definitely diagnosed by break-apart *TFE3* FISH probe previously, and 6 cases of these Xp11.2 tRCCs were identified as *ASPL-TFE3* RCC by dual-fusion FISH probe [30]. Their ages ranged from 3 to 64 years (mean age, 28y; median age, 26y) and the patients were identified as 14 females and 9 males (ratio of female to male patients was 1.6:1).

### TFE3 and cathepsin K IHC, Dual-Fusion FISH Probe in FFPE tissues

**Fig 1:**
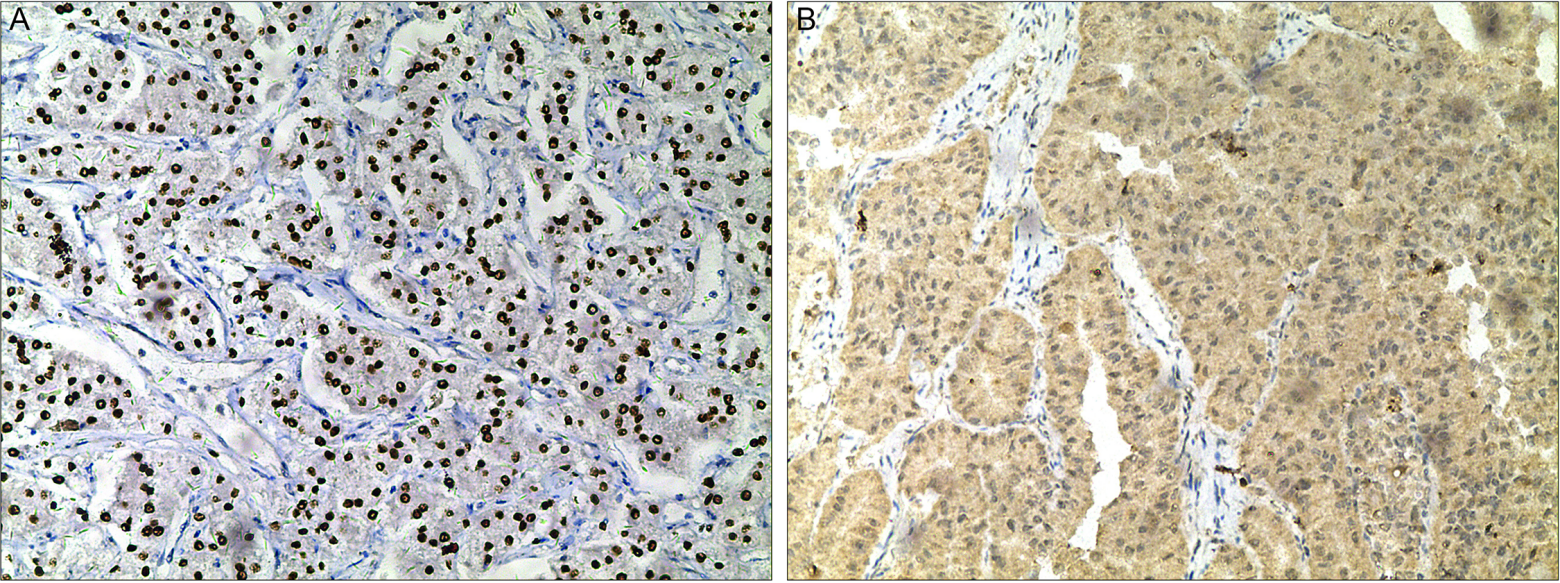
Images of immunohistochemical staining for *PRCC-TFE3* RCC. **(A) Strong nuclear immunostaining for TFE3 protein (×100); (B) The neoplastic cells showed diffuse cytoplasmic labeling for cathepsin K (×100).** RCC, renal cell carcinoma; TFE3, transcription factor E3; H&E, hematoxylin and eosin.

TFE3 and cathepsin K IHC were performed on the 23 Xp11.2 tRCCs and 40 non- Xp11.2 tRCCs. For TFE3, 22/23 Xp11.2 tRCCs and 3/40 non- Xp11.2 tRCCs showed moderately (2+) or strongly (3+) positive staining, and all *PRCC-TFE3* and *ASPL-TFE3* RCCs were positive (Fig 1A). Of the 23 Xp11.2 tRCCs, 16 cases demonstrated moderately (2+) or strongly (3+) positive staining for cathepsin K (include 7 *PRCC-TFE3* RCCs) (Fig 1B) and the remaining 7 cases were negative (include 6 *ASPL-TFE3* RCCs). All 40 non- Xp11.2 tRCCs were negative for cathepsin K.

**Fig 2:**
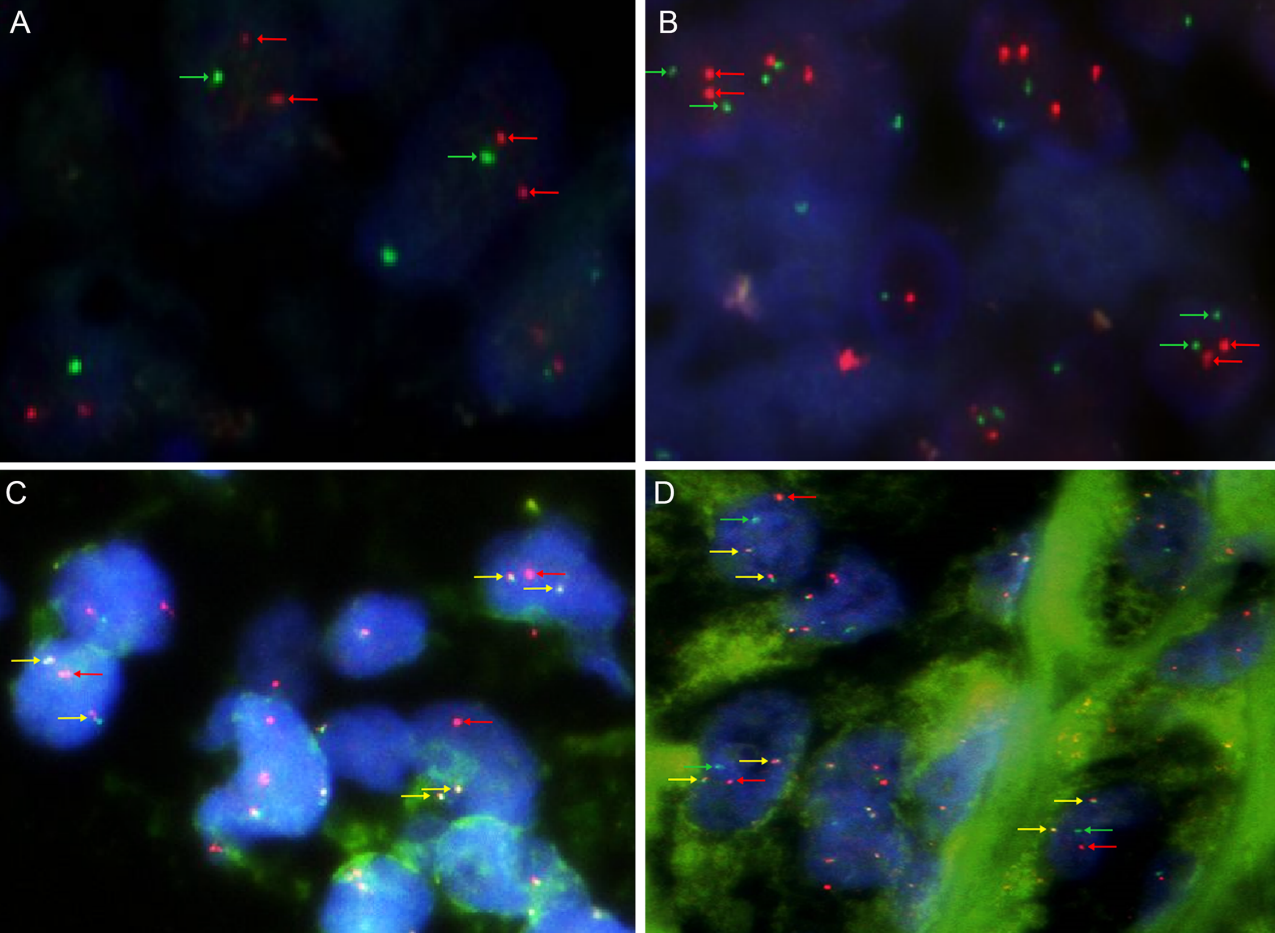
Images of the *PRCC-TFE3* Dual-Fusion FISH assay. **(A) Photomicrograph showed a pair of split red signals and a green signal (negative result, red and green arrows) in each nucleus in male (×1000); (B) Photomicrograph showed a pair of split red signals and two split green signals (negative result, red and green arrows) in each nucleus in female (×1000); (C) A positive result included 2 fused and 1 red signals (yellow and red arrows) in each nucleus in male, indicating that *PRCC-TFE3* fusion genes have been formed (×1000); (D) A positive result of female patient showed 2 fused, 1 green and 1red signals (yellow, green and red arrows) in each nucleus (×1000).** FISH, fluorescence in situ hybridization.

To identify the *PRCC-TFE3* fusion gene, *PRCC-TFE3* dual-fusion FISH assay was implemented on these Xp11.2 tRCCs, clear renal cell carcinomas and papillary renal cell carcinomas. Seven cases were diagnosed as *PRCC-TFE3* RCC, with 2 fusion signals emerged, but the other 16/23 cases and control groups were negative. Two cases (case 1 and 7) of the *PRCC-TFE3* RCCs were confirmed by PCR experiment previously [30]. Male and female patients had different signal patterns. In male patients, a positive result included 2 fused and 1 red signals (1R2F) (Fig 2C), but the positive result of female patients showed 2 fused, 1 green and 1red signals (1GR2F) (Fig 2D).

### Clinicopathologic features of *PRCC-TFE3* RCC

**Fig 3:**
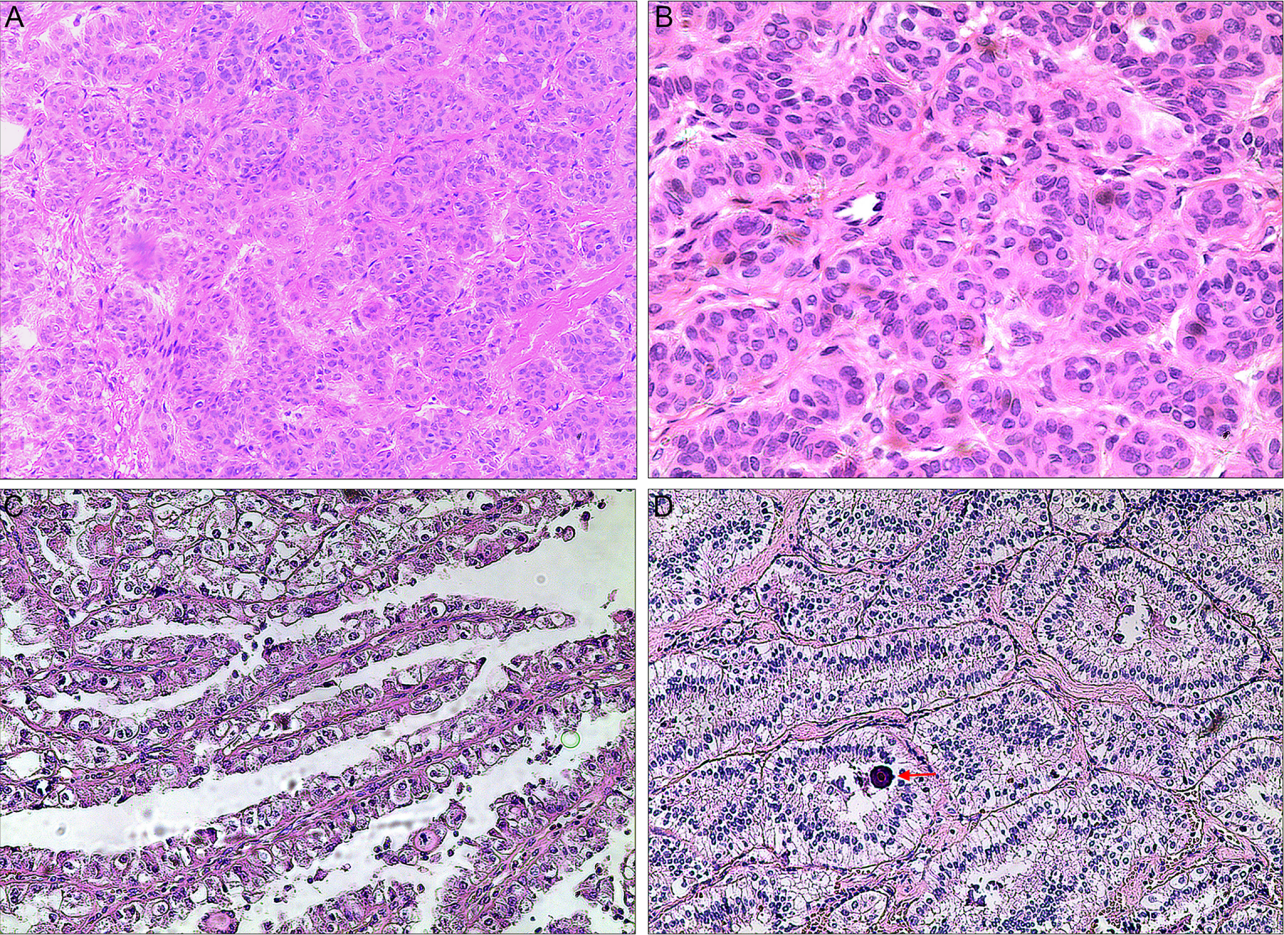
Images of microscopic morphology for *PRCC-TFE3* RCC. **(A) Photomicrograph showed solid nested architecture composed of compactly arranged, eosinophilic cells (H&E,×100); (B) Tumor cells showed irregular nuclei, inconspicuous nucleoli, eosinophilic cytoplasm, and indistinct cell borders (H&E,×200); (C) Photomicrograph showed histopathology of neoplasm containing clear to eosinophilic cells with voluminous cytoplasm arranged in a papillary pattern(H&E,×100); (D) Photomicrograph showed acinar pattern composed of compactly arranged, clear to slightly eosinophilic cells, and a psammoma body(arrow)(H&E,×100);** RCC, renal cell carcinoma; H&E, hematoxylin and eosin.

Seven cases were finally confirmed as *PRCC-TFE3* RCC by the dual-fusion FISH probe. Of the 7 *PRCC-TFE3* RCC patients, 3 were male and 4 female and median age was 30 years (22y to 64y). Two patients (cases 2 and 3) presented with hematuria and the remaining 5 patients were asymptomatic and discovered fortuitously. Nephrectomy was performed in all cases except case 6 who was treated by nephron-sparing surgery (tumor size was 3 cm). Mean size of the tumor was 6.1 cm (3 to 12 cm). Postoperative AJCC staging showed that 5 cases were classified as stage I, while the other 2 cases were classified as stage III. Mean follow-up was 34.6 months (5 to 76 mo). Five of the stage I cases received satisfactory results, except for case 1 who was diagnosed with lung metastasis at the 11th postoperative month. However, the 2 stage III patients (case 4 and 5) who received surgery and postoperative adjuvant molecular-targeted therapy developed local recurrence, and they received secondary surgeries in our hospital to resect recurrent lesions, with no disease progression until the last follow-up. Regarding pathologic morphology, the architecture of this tumor was predominantly nested and focal papillary or acinar, including clear or little eosinophilic cytoplasm, and psammoma bodies could be found in 2 cases (Fig 3). Data of the 7 cases, including the clinicopathologic features, TFE3 and cathepsin K IHC, *PRCC-TFE3* dual-fusion FISH assay, are summarized in Table 1.

**Table 1:** The data of clinicopathologic features, TFE3 and cathepsin K IHC, *PRCC-TFE3* FISH assay of *PRCC-TFE3* renal cell carcinomas.

Case	Age(years)/Sex	Symptom	Operation	Tumor size(cm)	ACJJ stage	TFE3 IHC	cathepsin K IHC	*PRCC-TFE3* dual-fusion FISH	Follow-up(months) and outcome
1	35/M	Symptomless	LRN	6	pT1bN0M0,Ⅰ	++	++	1R2F	52, Lung metastasis in 11 months, stable now.
2	22/F	Gross hematuria	LRN	5	pT1bN0M0,Ⅰ	+++	++	1G1R2F	40,Normal
3	25/F	Gross hematuria	LRN	3.5	pT1aNxM0,Ⅰ	++	+++	1G1R2F	15,Normal
4	45/F	Symptomless	ORN	12	pT3aN0M0,Ⅲ	+++	++	1G1R2F	32, Recur in 12 months
5	30/F	Symptomless	LRN	9.5	pT3aN0M0,Ⅲ	+++	+++	1G1R2F	22, Recur in 14 months
6	64/M	Symptomless	LNSS	3	pT1aN0M0,Ⅰ	++	+++	1R2F	5,Normal
7	26/M	Symptomless	ORN	3.7	pT1aN0M0,Ⅰ	+++	+++	1R2F	76,Normal

LRN: Laparoscopic radical nephrectomy; ORN: Open radical nephrectomy; LNSS: laparoscopic nephron-sparing surgery; IHC: immunohistochemistry; FISH: fluorescence in situ hybridization; TFE3, transcription factor E3.

## Discussion

Although *PRCC-TFE3* RCC is the first reported Xp11.2 tRCC, because of less clinicopathologic data, distinctive histopathologic characterization, recommended diagnostic criteria, and standardized therapeutic strategy of *PRCC-TFE3* RCC has yet to be established. As is well-known, accurate and concise diagnosis is crucial for treatment, and also for basic research. Relying only upon pathologic morphology and IHC, the subtypes of Xp11.2 tRCC cannot be discriminated. Genetic approaches, such as RT-PCR and FISH assay, are available to identify the type of genetic changes in tumor cells[13], but RT-PCR usually requires high quality RNA (fresh frozen samples). For FFPE archival sections, it has been demonstrated that FISH assay is an effective method for identifying gene translocation. Therefore, we performed this FISH probe on paraffin-embedded tissue to distinguish *PRCC-TFE3* RCC.

The design strategy in this assay imitates the *ASPL-TFE3* dual-fusion probe: BACs cover the entire *PRCC* and *TFE3* genes [30]. Two fusion signals emerge simultaneously in 1 nucleus, indicating that *PRCC-TFE3* fusion genes have been formed. In this study, 7 cases were diagnosed as *PRCC-TFE3* RCC using the probe. The result of FISH assay on 2 *PRCC-TFE3* RCCs was in accordance with the PCR result, which validated the efficacy of the *PRCC-TFE3* dual-fusion probe. Argani et al also analyzed *PRCC-TFE3* RCC by FISH assay to establish their *TFE3* fusion gene partner, but what they employed were the *TFE3* and *PRCC* break-apart FISH probes [11]. They detected the separation of *TFE3* gene to diagnose Xp11.2 tRCC first, followed by using *PRCC* break-apart probe to identify *PRCC-TFE3* RCC.

Morphologically, *PRCC-TFE3* RCC may be associated with differing features. Argani et al and subsequently Rao et al’s studies found that this tumor always had a nested(solid, alveolar, acinar, or tubular) pattern with foci of papillary architecture consisted by compact cells with less abundant cytoplasm and fewer psammoma bodies compared with *ASPL-TFE3* RCC[28, 33]. In our series, 7 cases had nested and focal papillary or acinar architecture and tumor cells included clear or little eosinophilic cytoplasm with only 2 cases to be found psammoma body, which was consistent with published data. However, this type of gene fusion-associated neoplasm cannot be validated by this distinction, which is not suitable for all cases. Immunohistochemically, except for molecular genetic analysis, nuclear reactivity of TFE3 protein is the most sensitive and specific method for *TFE3* rearrangement-associated neoplasm [8]. But TFE3 IHC in diagnosing Xp11.2 tRCC may lead to false-positive or false-negative results [34, 35]. Nuclear *TFE3* labeling can also appear in *ASPL-TFE3* RCC, *PSF-TFE3* RCC and other subtypes [8]. Our data showed that 22/23 Xp11.2 tRCCs, all 7 *PRCC-TFE3* RCCs and 3/40 non- Xp11.2 tRCCs were positive for TFE3 IHC, which indicated the meaninglessness of TFE3 IHC in diagnosing *PRCC-TFE3* RCC. Cathepsin K, a lysosomal papain-like cysteine protease which affects the bone reabsorption and remodeling [21], is always detected by IHC in MiT family tRCCs but not in other more common RCC subtypes. Interestingly, cathepsin K is distinguishingly expressed in Xp11.2 tRCC relying upon the fusion partner of the *TFE3* gene. For example, in Martignoni et al’s study [22], 8 *ASPL-TFE3* RCCs entirely did not express cathepsin K. Nevertheless, 12 of 14 *PRCC-TFE3* RCCs and all 18 alveolar soft part sarcomas were positive for this protein. Argani et al found that the Xp11.2 tRCCs with the *SFPQ-TFE3*, *NONO-TFE3*, *DVL2-TFE3*, and *ASPL-TFE3* gene fusions were almost cathepsin K negative by IHC, whereas a majority of *PRCC-TFE3* RCCs and 6 Xp11.2 tRCCs with unknown fusion partner were cathepsin K positive [11].The result of our study showed that seven *PRCC-TFE3* RCCs exhibited positive staining for cathepsin K but all of the *ASPL-TFE3* RCCs were negative, which was consistent with the findings of Martignoni et al. This diversity could be due to the functional differences between the fusion variants. However, the detailed molecular mechanism has not been well defined, which need further research.

It was reported that Xp11.2 tRCC typically had a predominance of females and affects young adults less than 45 years of age [36]. However, in *PRCC-TFE3* RCC, the gender diversity has not been proven and the frequency in adults is underestimated. In the current study, the sex ratio was three to four and case 6 was confirmed in a 64-year-old. There had been reported a series of cases who were older than 45 years [28, 37, 38]. We showed 2 patients presented with hematuria, whose tumor size was not the largest. *ASPL-TFE3* RCC was previously found to present at more advanced stage, poor prognosis and more likely to present with regional lymph node metastasis than the *PRCC-TFE3* RCC [29, 39]. However, we reported here one case in low stage developed metastasis (lung) 11month after resection, whereas the condition of this patient was fortunately stable with 52 month of follow up. Another two cases with large tumor size (stage III) developed local recurrence approximately 1 year after surgery. According to Argani et al’s studies, some cases of *PRCC-TFE3* RCC presented with metastatic disease and recurrence [11, 28]. Ellis et al observed that node-positive but non-metastatic cases with *PRCC-TFE3* RCC tended to have a worse outcome than those with *ASPL-TFE3* RCC [29]. But regional nodal metastasis was not found in these *PRCC-TFE3* RCC cases of our study. Differentiating the *PRCC-TFE3* RCC from other types is necessary for patient management.

At the molecular level, oncogenic mechanism of *TFE3* in Xp11.2 tRCC is driven by the different gene fusion partners, whereas it has not been well defined. It has been reported that the *TFE3* gene fusion partners owning strong promoters cause overexpression of the chimeric protein. Thereinto, *PRCC-TFE3* acts as an aberrant transcription factor leading to strong nuclear labeling for TFE3 by IHC. About the function of *PRCC*, Skalsky et al’s results suggest that it was a component of the pre-mRNA splicing complex [40]. When the *PRCC* gene is fused to *TFE3*, the function of splicing factor is disordered. *PRCC-TFE3* may also influence the transactivation capacity of the transcription factor and perturb mitotic checkpoint control. It has been reported that *PRCC-TFE3* was an approximately threefold stronger transactivator than native *TFE3*, causing the change of genetic regulation [41]. Weterman et al found that *PRCC-TFE3* fusion protein could impair the interaction of *PRCC* and MAD2B, sequentially disrupting the mitotic checkpoint in RCC [42]. The carcinogenic mechanism of *PRCC-TFE3* gene translocation in RCC is not yet clear, but it is crucial for the therapy of this carcinoma.

## Conclusions

In summary, *PRCC-TFE3* RCC is a rare tumor belonging to Xp11.2 tRCC. We developed a dual-fusion FISH assay as a relatively rapid and precise method to identify this type carcinoma in paraffin-embedded tissue. Furthermore, our study also characterized the morphologic, immunohistochemical and clinical features of the 7 *PRCC-TFE3* RCCs.

## Supporting information

S1 Table The data of clinicopathologic features, TFE3 and cathepsin K IHC, PRCC-TFE3 FISH assay of Xp11.2 translocation renal cell carcinomas. (DOCX)

S1 Fig The result of RT–PCR of PRCC-TFE3 RCC in previous study. A targeted PRCC-TFE3 transcript was in lane 1; lanes 2 was negative RT–PCR results of ASPL-TFE3; lanes 3 was clear cell RCC.
(DOCX)
