# Supplementary material for: PRCC-TFE3 dual-fusion FISH assay: A new method for identifying PRCC-TFE3 renal cell carcinoma in paraffin-embedded tissue
Source: PLoS One. 2017 Sep 26;12(9):e0185337. doi: 10.1371/journal.pone.0185337 (PMC5614571; doi:10.1371/journal.pone.0185337)
Supplement: S1 Table — (DOCX) [file pone.0185337.s001.docx]

**S1 Table. The data of clinicopathologic features, TFE3 and cathepsin K IHC, *PRCC-TFE3* FISH assay of Xp11.2 translocation renal cell carcinomas.**

| Case | Age(years)/Sex | Symptom | Operation | Tumor size(cm) | ACJJ stage | TFE3 IHC | cathepsin K IHC | PRCC-TFE3 dual-fusion FISH | Follow-up(months)  and outcome |
| --- | --- | --- | --- | --- | --- | --- | --- | --- | --- |
| 1 | 35/M | Symptomless | LRN | 6 | pT1bN0M0,Ⅰ | ++ | ++ | 1R2F | 52, Lung metastasis in 11 months, stable now. |
| 2 | 22/F | Gross hematuria | LRN | 5 | pT1bN0M0,Ⅰ | +++ | ++ | 1G1R2F | 40,Normal |
| 3 | 25/F | Gross hematuria | LRN | 3.5 | pT1aNxM0,Ⅰ | ++ | +++ | 1G1R2F | 15,Normal |
| 4 | 45/F | Symptomless | ORN | 12 | pT3aN0M0,Ⅲ | +++ | ++ | 1G1R2F | 32, Recur in 12 months |
| 5 | 30/F | Symptomless | LRN | 9.5 | pT3aN0M0,Ⅲ | +++ | +++ | 1G1R2F | 22, Recur in 14 months |
| 6 | 64/M | Symptomless | LNSS | 3 | pT1aN0M0,Ⅰ | ++ | +++ | 1R2F | 5,Normal |
| 7 | 26/M | Symptomless | ORN | 3.7 | pT1aN0M0,Ⅰ | +++ | +++ | 1R2F | 76,Normal |
| 8 | 25/M | Gross hematuria,  Flank pain | LRN | 7.1 | pT2aN0M0, Ⅱ | +++ | - | 1G2R | 20, Normal |
| 9 | 21/F | Symptomless | LRN | 4 | pT1aN0M0, Ⅰ | ++ | - | 2G2R | 62, Normal |
| 10 | 7/M | Gross hematuria | ORN | 10 | pT4N1M0, Ⅳ | +++ | - | 1G2R | 106, Normal |
| 11 | 36/F | Gross hematuria | ORN+VCTER | 8.6 | pT3cN1M0, Ⅲ | +++ | + | 2G2R | 35, Died of liver metastasis |
| 12 | 38/M | Gross hematuria,  Flank pain | LRN | 3 | pT1aN0M0, Ⅰ | +++ | + | 1G2R | 30, Normal |
| 13 | 39/F | Symptomless | ORN+VCTER | 13 | pT3bN1M0, Ⅲ | +++ | - | 2G2R | 27, Died of liver and brain metastasis |
| 14 | 19/F | Symptomless | LRN | 5 | pT1bN0M0, Ⅰ | ++ | ++ | 2G2R | 26, Normal |
| 15 | 26/M | Symptomless | LNSS | 3.7 | pT1aN0M0, Ⅰ | ++ | +++ | 1G2R | 20, Normal |
| 16 | 3/F | Gross hematuria | ORN | 4 | pT1aN1M0, Ⅲ | +++ | +++ | 2G2R | 73, Normal |
| 17 | 11/F | Gross hematuria,  Abdominal mass | ORN | 5.6 | pT1bN0M0, Ⅰ | ++ | + | 2G2R | 90, Lost |
| 18 | 29/M | Symptomless | LNSS | 3.5 | pT1aN0M0, Ⅰ | +++ | ++ | 1G2R | 12, Normal |
| 19 | 24/F | Symptomless | LRN | 3.9 | pT1aN0M0, Ⅰ | +++ | ++ | 2G2R | 42, Normal |
| 20 | 40/M | Symptomless | LRN | 3.9 | pT1aN0M0, Ⅰ | ++ | ++ | 1G2R | 39, Normal |
| 21 | 51/F | Symptomless | LNSS | 5 | pT1bNxM0, Ⅰ | +++ | +++ | 2G2R | 55, Normal |
| 22 | 26/F | Gross hematuria | LRN | 5 | pT1bN0M0, Ⅰ | +++ | +++ | 2G2R | 98, Normal |
| 23 | 27/F | Gross hematuria,  Flank pain | LRN | 6 | pT1bN0M0, Ⅰ | - | ++ | 2G2R | 55, Normal |

LRN: Laparoscopic radical nephrectomy; ORN: Open radical nephrectomy; LNSS: laparoscopic nephron-sparing surgery; VCTER: Vena cava tumor embolus resection; IHC: immunohistochemistry; FISH: fluorescence in situ hybridization; TFE3, transcription factor E3.
