# Supplementary material for: PRCC-TFE3 dual-fusion FISH assay: A new method for identifying PRCC-TFE3 renal cell carcinoma in paraffin-embedded tissue
Source: PLoS One. 2017 Sep 26;12(9):e0185337. doi: 10.1371/journal.pone.0185337 (PMC5614571; doi:10.1371/journal.pone.0185337)
Supplement: S1 Fig — A targeted PRCC-TFE3 transcript was in lane 1; lanes 2 was negative RT–PCR results of ASPL-TFE3; lanes 3 was clear cell RCC. (DOCX) [file pone.0185337.s002.docx]

**S1 Fig. The result of RT–PCR of PRCC-TFE3 RCC in previous study.**

A targeted PRCC-TFE3 transcript was in lane 1; lanes 2 was negative RT–PCR results of ASPL-TFE3; lanes 3 was clear cell RCC.**
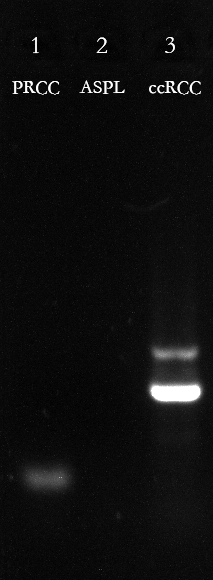
**
